# Feasibility and Performance Test of a Real-Time Sensor-Informed Context-Sensitive Ecological Momentary Assessment to Capture Physical Activity

**DOI:** 10.2196/jmir.5398

**Published:** 2016-06-01

**Authors:** Genevieve Fridlund Dunton, Eldin Dzubur, Stephen Intille

**Affiliations:** ^1^ Department of Preventive Medicine University of Southern California Los Angeles, CA United States; ^2^ College of Computer and Information Science & Dept. of Health Sciences Bouvé College of Health Sciences Northeastern University Boston, MA United States

**Keywords:** mobile phones, ecological momentary assessment, accelerometer, physical activity

## Abstract

**Background:**

Objective physical activity monitors (eg, accelerometers) have high rates of nonwear and do not provide contextual information about behavior.

**Objective:**

This study tested performance and value of a mobile phone app that combined objective and real-time self-report methods to measure physical activity using sensor-informed context-sensitive ecological momentary assessment (CS-EMA).

**Methods:**

The app was programmed to prompt CS-EMA surveys immediately after 3 types of events detected by the mobile phone’s built-in motion sensor: (1) Activity (ie, mobile phone movement), (2) No-Activity (ie, mobile phone nonmovement), and (3) No-Data (ie, mobile phone or app powered off). In addition, the app triggered random (ie, signal-contingent) ecological momentary assessment (R-EMA) prompts (up to 7 per day). A sample of 39 ethnically diverse high school students in the United States (aged 14-18, 54% female) tested the app over 14 continuous days during nonschool time. Both CS-EMA and R-EMA prompts assessed activity type (eg, reading or doing homework, eating or drinking, sports or exercising) and contextual characteristics of the activity (eg, location, social company, purpose). Activity was also measured with a waist-worn Actigraph accelerometer.

**Results:**

The average CS-EMA + R-EMA prompt compliance and survey completion rates were 80.5% and 98.5%, respectively. More moderate-to-vigorous intensity physical activity was recorded by the waist-worn accelerometer in the 30 minutes before CS-EMA activity prompts (M=5.84 minutes) than CS-EMA No-Activity (M=1.11 minutes) and CS-EMA No-Data (M=0.76 minute) prompts (*P*’s<.001). Participants were almost 5 times as likely to report going somewhere (ie, active or motorized transit) in the 30 minutes before CS-EMA Activity than R-EMA prompts (odds ratio=4.91, 95% confidence interval=2.16-11.12).

**Conclusions:**

Mobile phone apps using motion sensor–informed CS-EMA are acceptable among high school students and may be used to augment objective physical activity data collected from traditional waist-worn accelerometers.

## Introduction

Surveillance, epidemiological, and intervention studies typically use either retrospective self-report measures or objective sensor-based monitors (eg, accelerometers, heart rate devices) to capture physical activity behavior in youth [1-3]. Retrospective self-report methods ask participants to recall levels and types of activities performed over the past few days, weeks, or year [4]. As such, they can be vulnerable to various types of reporting errors and biases [5]. Although objective activity monitors may yield more valid measures of physical activity [6] and are being deployed in large-scale surveillance studies with children and adolescents [1], they may result in significant amounts of missing data due to device nonwear [7,8]. Participants forget to wear or carry monitors, and they remove monitors when they do not want to or cannot wear them. Often, these data are not missing at random because youth remove the monitors during physical activity bouts such as swimming or those bouts involving physical contact with others (eg, football, soccer), which can result in biased activity estimates [9].

In addition to high rates of device nonwear, objective activity monitors are unable to capture descriptive and contextual information about physical activity and sedentary behavior. Accelerometers and other objective activity monitors typically assess the intensity and duration of physical body movement [10,11]. Yet, most devices cannot measure the type and purpose of the activity, emotional responses to the activity, where and with whom the activity occurred, and other cognitive and motivational factors. Information about these real-time psychological and environmental correlates of physical activity has growing importance for the development of just-in-time adaptive interventions to encourage physical activity at the point of decision-making during adolescents’ everyday lives [12,13].

Methods of physical activity assessment that collect real-time self-report data about activities and contexts during key moments of the day, such as when an accelerometer is removed, or immediately after a bout of physical activity, have the potential to yield information that an accelerometer cannot. Ecological momentary assessment (EMA) is one such real-time self-report data capture method, which elicits responses to electronic surveys throughout the course of daily life [14,15]. EMA has many advantages over retrospective self-report measures such as reducing memory errors and biases and collecting more ecologically valid assessments in one’s natural environment [16-18]. Standard EMA uses *interval-contingent* sampling to trigger surveys at preset times (eg, at 8 am and 12 noon everyday), *signal-contingent* sampling to trigger surveys at random times throughout the day, or *event-contingent* sampling to trigger surveys during or after a predetermined behavior such as a bout of physical activity [16]. However, these sampling strategies suffer from a number of limitations. Interval- and signal-contingent sampling strategies often fail to capture less common behavior, such as physical activity, as it is occurring. Event-contingent sampling requires the participant to self-initiate surveys during or after particular behavior, which is prone to delayed reports and the failure to report events altogether. CS-EMA is an innovative strategy that addresses these problems by automatically triggering survey prompts at opportune times based on detected information from internal or external sensors to collect real-time information about key behaviors, events, or contexts [19].

This study tested a mobile phone app called Mobile Teen that implemented CS-EMA by performing real-time analysis on data gathered about the mobile phone’s movement from the mobile phone’s built-in motion sensor. The app detected major transitions in type of mobile phone movement and subsequently triggered real-time electronic surveys that collected self-report information about recent physical activity and sedentary behavior. A growing number of studies have measured physical activity using native mobile features with varying levels of accuracy [20,21]. The goals of this study were to examine the feasibility, performance, accuracy, and utility of the Mobile Teen EMA app for capturing information about physical activity behaviors and contexts in adolescents. Specially, the objectives were to: (1) describe EMA survey compliance, response latency, and completion time and rates, in addition to how the mobile phone was carried; (2) evaluate the performance of the CS-EMA prompting features by examining differences in reported and objectively measured activity levels across the different EMA prompt types; (3) describe the extent to which EMA provides data about activity during periods of waist-worn accelerometer nonwear; and (4) describe contextual characteristics of EMA-reported sports and exercise episodes.

## Methods

### Mobile Teen App

The Mobile Teen app was designed for mobile phones running the Android operating system. The software was written in Java and targeted Android versions 2.3.3 to 4.3, the versions available at the time of the research. The Mobile Teen app was programmed to conduct sensor-informed CS-EMA by using the mobile phone’s built-in motion sensor to automatically detect periods of (1) Activity (15+ minutes of high-intensity activity followed by 10+ minutes of low-intensity activity); (2) No-Activity (60+ minutes of low-intensity activity followed by 2+ minutes of moderate-intensity activity); and (3) No-Data (10+ minutes of no activity data followed by 1+ minutes of some activity data). The app then used these sensor-informed movement transition cues to trigger real-time CS-EMA self-report surveys measuring the type and purpose of activity previously performed, enjoyment of that activity, and social and physical features of the activity setting. A more detailed description of the design and development of the Mobile Teen app is available elsewhere [22].

### Participants and Recruitment

The study sample consisted of low-to-middle–income adolescents in grades 9 to 12. Participants were recruited through an urban Los Angeles area high school using informational fliers, posters, and classroom visits. Inclusion criteria were as follows: (1) student in grade 9 to 12 enrolled at the participating high school, (2) able to comprehend written English, (3) no health or physical limitations that prohibit regular physical activity, and (4) regular use of an Android or global system for mobile communication (GSM)-based mobile phone with service provided by AT&T or T-Mobile on a standard mobile phone contract. For adolescents who did not own an Android mobile phone, the restriction to having a GSM-based mobile provider (AT&T or T-Mobile) was made so that their personal phone subscriber identity module (SIM) cards could be easily switched to temporary LG Nexus 4 mobile phones with the Mobile Teen app installed for the duration of the study. Doing so allowed participants to use the study mobile phone to make and receive calls and short message service (SMS) messages through personal mobile phone numbers. Adolescents who expressed interest in the study during a school visit were called by phone to be screened for eligibility and scheduled for an orientation session with research staff. Parental consent and child assent were obtained. Study procedures were approved by the Institutional Review Boards at the University of Southern California and Northeastern University.

### Procedures

Participants attended a 45-minute orientation session at the high school on a weekday afternoon. During this session, they completed a questionnaire and anthropometric assessments. Research staff assisted participants with either downloading the Mobile Teen app to their personal Android mobile phone or moving their SIM card from a personal mobile phone into a loaned Android study mobile phone with the Mobile Teen app preinstalled. They were also given instructions on how to complete EMA surveys. Over the next 28 days, participants were asked to wear the accelerometer and proceed with their daily routines as normal. Participants were asked to carry the mobile phone as usual (in their pockets, hands, purses, or bags) during waking hours. On 14 of those days (either the first 2 weeks or the second 2 weeks, as randomly assigned), they received EMA survey prompts several times per day during nonschool waking hours (3-9 pm on weekdays and 7 am-9 pm on weekend days). Research staff members contacted participants twice by phone during the 28-day monitoring period to encourage compliance and address technical issues. Participants received up to $180 for completing the study.

### Measures

#### Ecological Momentary Assessment

The Mobile Teen app triggered mobile phone sensor-informed CS-EMA prompts according to the 3 prompting rules described previously. In addition, the app triggered random (ie, signal-contingent) R-EMA prompts up to 3 times per day on weekdays and 7 times per day on weekend days. On receiving an auditory EMA prompt (a pleasant, but loud, 4-second chime), participants were instructed to stop their current activity and complete a short electronic survey question sequence using the touch screen of the mobile phone. Participants were allowed to set mobile phones to mute or vibrate to prevent interruption. If an EMA prompt occurred during an incompatible activity (eg, sleeping, bathing, or driving), participants were instructed to ignore it. If no entry was made, the app emitted up to 2 reminder prompts at 3-minute intervals. After this point, the electronic EMA survey became inaccessible until the next prompting opportunity. To avoid excessive prompting, the app enforced a 30-minute gap between all prompts.

**Figure 1:**
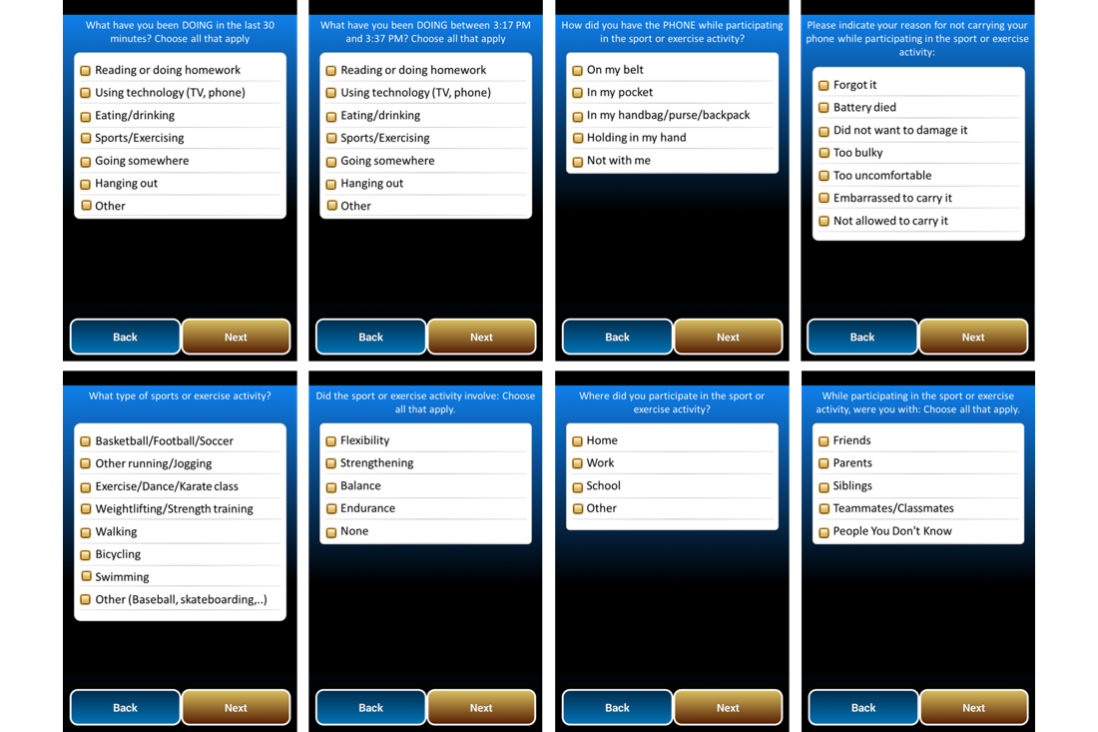
Mobile Teen app EMA screenshots, which include 8 sample survey items from the Mobile Teen app’s EMA interface. The items in the top row query (left to right) activity type during random EMA prompts, activity type during a context-sensitive EMA prompt, how the phone was carried, and reasons for not carrying the device (if it was not carried). Items on the bottom row (left to right) query specific type of sports or exercise, fitness skill involved, physical context, and social context.

EMA items assessed major activity types, methods of carrying the mobile phone, reasons for not carrying the mobile phone, and other psychological and contextual factors related to behavior (See Figure 1). CS-EMA prompts asked, “What have you been doing between (start time) and (stop time)?” with times inserted by the app based on information from the built-in mobile phone motion sensor. Alternatively, the R-EMA question sequence began with the *activity type* question, “What have you been doing in the last 30 minutes?” For both the CS-EMA and R-EMA activity-type questions, a response structure was used where participants could indicate 1 or more activities (ie, “choose all that apply”) that were performed during the time period. Response options include, “*Reading or doing homework; Using technology (TV, phone); Eating/Drinking; Sports/Exercising; Going somewhere; Hanging out; Other*.” If *Other* was selected, an extra question listed additional options including, “*Doing chores/Cooking; Showering/Bathing; Sleeping; Working/Part-time job; Getting ready for something; Shopping; Getting dressed; Class/School; Playing with children; Playing catch; Waiting; Doing something else.”* If *Sports/Exercising* was reported as an activity type, a follow-up question asked about the specific type of sports or exercise activity performed (eg, *Basketball/Football/Soccer, Bicycling, Other Running/Jogging*, *Exercise/Dance/Karate class, Weightlifting/Strength training).*

For each type of activity reported, participants were asked to indicate how long it lasted (in minutes), the body position (eg, *Lying down, Sitting, Standing*), how the mobile phone was carried (eg, *On my belt, In my pocket, Not with me*) and the reason for not having the mobile phone with them (eg, *Forgot it, Did not want to damage it, Too uncomfortable*). If *Sports/Exercising* was reported as an activity type, a unique question branching sequence was initiated that asked about type of fitness skill involved (eg*, Flexibility, Strengthening, Endurance*), extra weight carried (eg, *None, Less than 5 lbs, 5-10 lbs*), degree of incline (eg, *Mainly going uphill, Mainly going downhill, Mainly staying on flat ground*), the physical context (eg, *Home, Work, School*), the main purpose (eg, *Fun/Recreation; To get somewhere; For work, homework, or housework*), how enjoyable it was (eg, *Not at all, A little* *, Moderately*), and the social context (eg, *Alone or With Friends, Parents, Siblings*). The branching sequence pertaining to sports and exercise activities was only initiated in a randomly programmed 40% of applicable surveys as a method of limiting potential subject response burden.

#### Waist-Worn Accelerometer

The Actigraph, Inc, activity monitor provided an objective measure of physical activity. A combination of GT1M, GT2M, and GT3X models were used. The device was worn on the right hip attached to an adjustable belt. Activity monitors were not worn while sleeping, bathing, or swimming. A 30-second epoch was used. All accelerometer recordings were time-stamped to be linked with EMA data captured on the mobile phone. Outcome variables consisted of the total number of minutes of moderate-to-vigorous physical activity (MVPA) occurring within the 30-minute window before each EMA prompt. MVPA was defined using age-specific thresholds generated from the Freedson prediction equation (≥4 metabolic equivalents [METs]) [23]. Strings of continuous zero activity counts lasting 60 minutes or more were considered to be periods of waist accelerometer nonwear (with an allowance of up to 2 minutes with nonzero activity counts during that period) [1].

#### Body Mass Index

Research staff measured height and weight using an electronically calibrated digital scale (Tanita WB-110A) and professional stadiometer (PE-AIM-101) to the nearest 0.1 kg and 0.1 cm, respectively. Body mass index (BMI) was calculated (kg/m^2^) and converted to Centers for Disease Control and Prevention age- and gender-specific BMI percentiles.

#### Demographic

Participants’ age, gender, year in school, and ethnicity were assessed through a self-report paper-and-pencil questionnaire. Parents reported annual household income.

### Data Processing and Analyses

Descriptive statistics for demographic characteristics were calculated with individual participant as the unit of analysis (Level-2); all other descriptive statistics used occasions (ie, EMA prompts) as the unit of analysis (Level-1). Data were analyzed with multilevel modeling in Stata (version 14). Multilevel models adjust the standard errors for clustering of EMA prompts (Level-1) within people (Level-2) [24]. Between-subject (BS) and within-subject (WS) versions (ie, partitioning the variance) of the main effects were generated. The BS version represents the individual mean deviation from the grand mean, and the WS version represents deviation from one’s own mean at any given prompt [25]. Similarly, the BS and WS variation terms for binary predictors were created using grand mean-centering (ie, subtracting by the group mean proportion) and person mean-centering (ie, subtracting by the individual mean proportion) methods, respectively [26].

Descriptive statistics (ie, mean, standard deviations [SDs], frequencies, and percentages) were calculated to examine EMA compliance, response latency, and completion time and rates, as well as how the mobile phone was carried (objective #1). In addition, a series of multilevel logistic regressions were run to examine whether any demographic or temporal variables were associated with EMA compliance (binary outcome categorized as 0=unanswered prompt and 1=answered prompt), methods of carrying the mobile phone (binary outcome categorized as 1=not with me and 0=all else), and reason for not having the mobile phone with him or her (binary outcome categorized as 1=did not want to damage it and 0=all else).

Multilevel linear regression models were computed to compare EMA prompt types in terms of self-reported and objectively measured activity levels (objective#2). The models tested whether there were differences across the 4 EMA prompt types (ie, R-EMA, CS-EMA Activity, CS-EMA No-Activity, and CS-EMA No-Data) in terms of objective levels of MVPA (measured by the waist-worn Actigraph) in the 30 minutes before the prompt. In addition, multilevel logistic linear regression models tested whether there were differences across prompt types (ie, R-EMA, CS-EMA Activity, CS-EMA No-Activity, and CS-EMA No-Data) in terms of the likelihood of engaging in each type of activity (vs all other activities) in the time leading up to the EMA prompt (ie, 30-minute intervals before R-EMA prompts and automatically detected time intervals before CS-EMA prompts). Each type of activity was tested in a separate logistic regression model. The 4-level EMA prompt type served as the independent variable (with R-EMA designated as the reference group) for the linear and logistic regressions testing the second study objective. Models testing the likelihood of engaging in each type of activity across EMA prompt types and models examining objective MVPA across prompt types were also adjusted by the day of week, time of day, chronological day in study, age, and gender. Random intercept models were estimated. Descriptive statistics (ie, means, SDs, frequencies, and percentages) were run to describe the extent to which EMA provides data about activity during periods of waist-worn accelerometer nonwear (objective #3) and measures contextual characteristics of EMA-reported sports and exercise episodes (objective #4).

## Results

### Participant Characteristics

Of 248 participants who initially expressed interest in the study, 5 participants lost interest, 2 of them were excluded based on asthma history, 10 of them did not have mobile phones, 126 of them could not be reached for scheduling, and 37 of them canceled their scheduled appointments; the remaining 68 participants met the inclusion criteria and verbally agreed to join at a mobile phone screening interview. A total of 44 participants subsequently attended the scheduled orientation appointment with their parents and consented to participate. During the study period, 4 participants voluntarily dropped out, leaving a total of 40 participants who completed the study. One participant lost the waist-worn accelerometer, resulting in 39 participants with complete data for analysis. The analytic sample consisted of 21 girls and 18 boys between the ages of 14 and 18 (M=15.9 SD=1.2). Sixty-four percent reported Hispanic or Latino ethnicity, and all participants reported receiving free or reduced lunch at school. Table 1 summarizes further demographic characteristics of the sample.

**Table 1:** Demographic characteristics of participants (N=39).

Characteristics		N (%)
Gender		
	Male	18 (46.2)
	Female	21 (53.9)
BMI^a^ (mean, SD^d^)		24.58 (4.3)
Age (mean, SD)		15.90 (1.2)
Grade in school^b^		
	9	9 (23.7)
	10	11 (29.0)
	11	9 (23.7)
	12	9 (23.7)
Ethnicity^c^		
	White	7 (18.4)
	Black or African-American	1 (2.6)
	Hispanic/Latino	25 (65.8)
	Asian	4 (10.5)
	Other	1 (2.6)
Mobile phone platform		
	Google Android	27 (69.2)
	Apple iOS	12 (30.8)
Mobile phone device used	Loaned mobile phone	22 (56.4)
	Personal mobile phone	17 (43.6)

^a^BMI: body mass index.

^b^Information on grade in school was not reported for 1 participant.

^c^Ethnicity was not reported for 1 participant.

^d^SD: standard deviation.

**Table 2:** Ecological momentary assessment (EMA) prompts and compliance.

Variable	Total prompts	Average Compliance (%)^a^	SD^b^ (%)	Range (%)
All prompts	3,631	80.5	14.6	38.2-97.0
R-EMA^c,g^	2,830	79.4	14.3	35.6-97.6
CS-EMA^h^ activity^d^	40	87.8	28.5	0.0-100.0
CS-EMA No-Activity^e^	369	88.6	14.7	40.0-100.0
CS-EMA No-Data^f^	392	85.0	21.6	16.7-100.0

^a^Compliance (%) represents the number of EMA surveys answered divided by the number of EMA surveys prompted.

^b^SD: standard deviation

^c^Prompts occurring randomly within schedule intervals throughout the day.

^d^Prompts occurring after a 15-minute period of high-intensity activity followed by a 10-minute period of low-intensity activity.

^e^Prompts occurring after a 60-minute period of low-intensity activity followed by 2+ minutes of moderate-intensity activity.

^f^Prompts occurring after a 10-minute period of no activity data followed by a period of 1 minute of available data.

^g^R-EMA: random ecological momentary assessment.

^h^CS-EMA: context-sensitive ecological momentary assessment

### EMA Compliance

Descriptive statistics for R-EMA and CS-EMA compliance rates are summarized in Table 2. Compliance rate was defined as the number of answered EMA surveys divided by the number of EMA surveys prompted when the mobile phone was powered on and charged. Prompts consisted of audible or tactile feedback, unless the app was in silent mode, in which case, the app would appear on the screen if the mobile phone was unlocked, but it did not otherwise alert the participant. In total, across all the participants, CS-EMA No-Activity and CS-EMA No-Data prompts were more common than CS-EMA Activity prompts. Average compliance rates were higher for CS-EMA prompts (84.8%, standard error of the mean [SE]=2.2%) than R-EMA prompts (78.8%, SE=2.4%; z=3.67, *P*<.001). In addition, average EMA compliance was higher when children were wearing a waist accelerometer in the 30 minutes leading up to the EMA prompt (83.0%, SE=1.9%) than when not wearing the waist accelerometer during this period (75.5%, SE=2.6%; z=4.71, *P*<.001). Average compliance to EMA prompts generally decreased across the study period. Individuals were 4.7% less likely to respond to an EMA prompt on a given day in the study compared with the day before, (z=−4.8, *P*<.001). However, compliance increased across the day. Participants were 8.7% more likely to respond to an EMA prompt at any given hour in a day compared with the hour before, (z=5.32, *P*<.001). Participants identifying as Hispanic were 2.21 times as likely to respond to any given EMA prompt than those identifying as any other ethnicity (z=2.65, *P*<.01). EMA compliance rates were unrelated to age, gender, BMI, grade in school, and household income (P values > .05). In total, 784 R-EMA surveys (25.8%) were not prompted at all either due to the mobile phone being powered off at the time the EMA prompt was scheduled or due to unknown technical problems. Unprompted R-EMA surveys were not included in the calculation of compliance rates.

### EMA Response Latency and Completion Time and Rate

Overall, 2,862 R-EMA + CS-EMA surveys were completed (ie, all questions answered) out of the 2,907 survey prompts that were answered, yielding an EMA survey completion rate (once started) of 98.5%. A total of 2,372 (82.3%) completed EMA surveys were responded to after the first prompt, 318 surveys (11.0%) after the first reprompt (3 minutes later), and 193 surveys (6.7%) after the second reprompt (6 minutes later). EMA survey completion time was defined as the time lag between receiving an EMA prompt and finishing the last question on the survey for that EMA prompt. EMA prompts requiring one or more reprompts (n=511) were not included in the calculation of completion time. The app was designed to time out if a survey was not completed within 15 minutes after the prompt. A total of 22 surveys that timed out for this reason were not included in the analysis of survey completion time. On average, surveys that were completed without reprompting were completed in 53.2 seconds (SD=47.27, range: 8-408). There were no differences in EMA completion time by age, gender, ethnicity, day of week, and time of day. However, participants completed surveys 1.61 seconds faster (confidence interval [CI]: 1.14-2.07) per chronological day in the study, (z=−6.79, *P*<.001; i.e., 20 seconds faster by the end of the study).

### Methods of Carrying the Mobile Phone

Analyses examined how the mobile phone was carried by participants because movement of the mobile phone was designed to trigger CS-EMA prompting. Participants were instructed to carry the mobile phones as they normally would. That is, they were not asked to carry the mobile phone in a special way (eg, on the hip), to investigate how normal mobile phone usage could drive the sensor-informed CS-EMA feature of the Mobile Teen app even if the mobile phone is carried in a pocket or bag. Although a sensor not on the body would not capture body motion directly, large changes in motion (or lack of motion) could align with large changes in activity patterns. EMA prompts asked how the mobile phone was carried during each type of activity reported. When participants reported engaging in 2 or more activities (n=476) during any given EMA prompt, only one of those activities was randomly selected for inclusion in analysis of how the mobile phone was carried. Table 3 summarizes the distribution of methods for carrying the mobile phone device by gender. As compared with boys, girls were more than 3 times as likely to have the mobile phone *Within reach* (ie, close by but not physically on their bodies) and less likely to carry the mobile phone in their pockets. Across boys and girls, reasons provided for not having the mobile phone (ie, *Not with me*) during some portion of the window of time on which they were reporting activities were forgetting it elsewhere (15.4%), the battery died (10.3%), not wanting to damage it (29.1%), feeling it was too bulky (6.8%), feeling it was too uncomfortable (9.4%), being too embarrassed to carry it (5.1%), and not being allowed to carry it (23.9%).

**Table 3:** Methods of carrying the mobile phone device by gender.

Method of carrying device^a^	Boys (%)	Girls (%)	OR^b^ (95% CI^c^)^d^
Carrying in pocket	47.0	17.0	0.15 (0.06-0.35)
Holding in hand	18.4	25.6	1.72 (0.70-4.18)
Carrying in bag or purse	3.5	6.8	2.12 (0.82-5.51)
On belt	1.1	1.2	1.15 (0.57-2.35)
Within reach	21.7	41.6	3.11 (1.54-6.25)
Not with me	5.2	8.8	1.82 (0.88-3.77)

^a^Dependent variable was coded as 1=method of carrying device, 0=all other methods.

^b^OR: odds ratio

^c^CI: confidence interval.

^d^Gender was coded as 1=female, 0=male. Results were generated from multilevel logistic linear regression models testing whether there were gender differences across methods of carrying the mobile phone immediately before the prompt. Each method of carrying the mobile phone was tested in a separate multilevel logistic regression model. Percentages (%) represent the adjusted margins generated from the statistical models.

Analyses further examined differences in reasons for not having the mobile phone (ie, *Not with me*) by time of day, day in the study, and other demographic factors. For each additional hour in a day, participants were twice as likely to report not having the phone because the battery died (odds ratio [OR]=2.04; 95% CI=1.06-3.93), and less likely to report not having the mobile phone because the mobile phone was too bulky compared with any other reason (OR=0.75; 95% CI=0.58-0.98). Furthermore, participants were less likely to report not being allowed to have the mobile phone, compared with any other reason, for each additional chronological day in the study (OR=0.62; 95% CI=0.43-0.89). Gender, age, grade in school, ethnicity, income, BMI, and day of the week were not related to reported reasons for participants not having the mobile phone (*P* values>.05). Participants were more likely to report having the mobile phone before answered CS-EMA Activity (92.2%) than CS-EMA No-Activity (64.8%; z=−2.70, *P*<.01) or CS-EMA No-Data (67.3%; z=−3.05, *P*<.01) type EMA prompts.

**Table 4:** Type of activity reported by ecological momentary assessment (EMA) prompt type.

	EMA prompt type								
Activity type^a^	R-EMA^b, c^	CS-EMA^d^ Activity^e^			CS-EMA No-Activity^f^			CS-EMA No-Data^g^		
	%	%	OR^i^(95% CI^j^)		%	OR (95% CI)		%	OR (95% CI)
Reading or doing homework	18.3	0.0	—		13.1	0.63 (0.44-0.90)		15.4	0.78 (0.50-1.22)
Using technology	24.6	2.9	0.08 (0.02-0.35)		29.2	1.30 (0.99-1.70)		16.9	0.59 (0.36-0.97)
Eating or drinking	10.0	19.3	2.18 (0.75-6.31)		8.3	0.81 (0.53-1.24)		9.9	0.99 (0.56-1.75)
Sports or exercise	3.7	10.2	3.66 (0.67-19.81)		7.8	2.54 (1.46-4.42)		4.6	1.30 (0.63-2.71)
Going somewhere	14.6	43.2	4.91 (2.16-11.13)		8.1	0.51 (0.33-0.78)		11.7	0.76 (0.47-1.24)
Hanging out	14.4	24.3	2.07 (0.90-4.76)		13.1	0.89 (0.61-1.30)		14.9	1.05 (0.66-1.67)
Sleeping	4.9	0	—		5.5	1.15 (0.64-2.07)		9.6	2.37 (1.29-4.35)
Other^h^	10.5	13.3	1.34 (0.50-3.57)		14.8	1.53 (1.07-2.20)		13.7	1.39 (0.87-2.22)

^a^Type of activity taking place during the time leading up to the EMA prompt (ie, 30-minute intervals before R-EMA prompts and automatically detected time intervals before CS-EMA prompts). Values in the table represent the proportion of the prompt type (column) reported in each activity type.

^b^R-EMA: random ecological momentary assessment.

^c^Prompts occurring randomly within scheduled intervals throughout the day.

^d^CS-EMA: context-sensitive ecological momentary assessment.

^e^Prompts occurring after a 15-minute period of high-intensity activity followed by a 10-minute period of low-intensity activity.

^f^Prompts occurring after a 60-minute period of low-intensity activity followed by 2+ minutes of moderate-intensity activity.

^g^Prompts occurring after a 10-minute period of no activity data followed by a period of 1 minute of available data.

^h^All other activities were recoded into the “Other” category. Results were generated from multilevel logistic linear regression models testing whether there were differences across prompt types (ie, R-EMA, CS-EMA Activity, CS-EMA No-Activity, and CS-EMA No-Data) in terms of the likelihood of engaging in each type of activity (vs all other activities) immediately before the prompt. Each type of activity was tested in a separate multilevel logistic regression model. EMA prompt type served as the independent variable with R-EMA designated as the reference group. Models were adjusted by day of week, time of day, chronological day in study, age, and gender. OR for WS effects are shown. There were no significant BS effects for any activity type (*P* values>.05; results not shown). Percentages (%) represent the adjusted margins generated from multiple independent statistical models controlling for the covariates, and therefore, the sum of a column may exceed 100% or fail to add up to 100%.

^i^OR: odds ratio.

^j^CI: confidence interval.

### Differences in Activity Levels by EMA Prompt Types

Results indicate that there were significantly more MVPA minutes recorded in the 30-minute window before CS-EMA Activity prompts (M=6.02, SE=0.74 minutes) than CS-EMA No-Activity (M=1.12, SE=0.48 minutes; z=−7.94, *P*<.001) and CS-EMA No-Data (M=0.77, SE=0.48 minutes; z=−7.68, *P*<.001) prompts. Table 4 compares EMA prompt types in terms of the type of activity reported to take place in the time leading up to the EMA prompt (ie, 30-minute interval before R-EMA prompts and automatically detected time intervals before CS-EMA prompts). When participants reported engaging in 2 or more activities (n=476) during any given EMA prompt, only one of those activities was randomly selected for inclusion in this analysis. Participants were almost half as likely to report reading or doing homework before CS-EMA No-Activity prompts compared to R-EMA prompts. Participants were more than twice as likely to report engaging in sports or exercise before CS-EMA No-Activity prompts compared to R-EMA prompts. Participants were almost 5 times as likely to report going somewhere (ie, active or motorized transit) before CS-EMA Activity than R-EMA prompts but were half as likely to report going somewhere before CS-EMA No-Activity than R-EMA prompts. Participants were twice as likely to report sleeping before CS-EMA No-Data prompts than R-EMA prompts. As compared with answered R-EMA prompts, participants were more likely to report using technology before CS-EMA No-Activity prompts and less likely to report using technology before CS-EMA No-Data and CS-EMA Activity prompts. Participants were one and a half times as likely to report other activities (eg, getting ready, working part-time) before CS-EMA No-Activity than R-EMA. There were no significant BS effects for any activity type.

### EMA Data Provided During Waist Accelerometer Nonwear Periods

Between the hours of 3 pm and 9 pm on weekdays, participants wore the waist accelerometer on an average of 4.09 (SD=2.16) hours per day (68.2% of the time). On 39 (10.3%) weekdays, waist-worn accelerometers were not worn at all (ie, less than 1 minute of total wear time). On weekend days between the hours of 7 am and 9 pm, participants wore the waist activity monitor on an average of 7.65 (SD=4.73) hours per day (54.6% of the time). On 23 (15.9%) weekend days, waist-worn accelerometers were not worn at all. Across all participants, there was a total of 885 answered EMA prompts (M=1.58, SD=2.30 per person per day) during periods when the waist accelerometer was not worn in the past 30 minutes. Of these prompts, 74.7% were EMA signal-contingent, 0.6% were CS-EMA Activity, 10.1% were CS-EMA No-Activity, and 14.5% were CS-EMA No-Data. Data on duration and type of self-reported activity (provided through answered R-EMA + CS-EMA) were summarized during waist accelerometer nonwear periods up to 60 minutes before each EMA prompt. EMA data provided an average of 31.9 (SD=54.10) minutes per person per day of activity data during waist accelerometer nonwear time (ie, that would have been missed if waist accelerometers alone were used). There was significantly more activity data per person per day provided by EMA during waist accelerometer nonwear periods on weekends (M=41.97, SE=6.08) than on weekdays (M=28.52, SE=5.32; z=3.06, *P*<.01). During waist accelerometer nonwear time periods, R-EMA + CS-EMA captured an additional 21 self-reported physical activity episodes (about 1 per child) that would have been missed using the waist-worn waist accelerometer alone. During waist accelerometer wear time periods, 20 bouts of activity were detected by CS-EMA Activity prompts. Seventeen bouts contained at least 10 minutes of light-intensity physical activity (ie, activity that does not meet MVPA thresholds), and only 3 had at least 10 minutes of MVPA. Of 58 bouts of MVPA detected by waist accelerometer that lasted longer than 15 minutes, 3 were detected by CS-EMA No-Activity, and 1 was detected by CS-EMA No-Data.

### Contextual Characteristics of EMA-Reported Sports and Exercise

In total, sports and exercise were reported during 108 of 2,795 (3.7%) answered R-EMA and CS-EMA prompts. At each prompt, participants reported the duration of each activity within the window queried by the EMA. Sports and exercise activities had an average reported duration of 38.7 (SD=17.6 min) minutes before the EMA prompt. On average, the 30-minute windows before R-EMA and CS-EMA prompts with reported sports and exercise had 1.80 (SD=3.81) minutes of MVPA and 3.37 (SD=3.90) minutes of sedentary activity (measured by the waist-worn activity monitor). While performing that exercise or sport, children reported with the following frequencies that the mobile phone was being worn on their belt (2.8%), in their pocket (31.1%), in their handbag, purse, or backpack (21.7%), or that they were holding it in their hand (14.2%). Conversely, during sports and exercise, it was reported that the mobile phone was within reach but not being worn on the participant (9.4%) and not with the participants (20.8%). Reasons reported for not having the mobile phone (ie, *Not with me*) during sports and exercise were as follows: the battery died (4.5%), not wanting to damage it (59.1%), feeling it was too bulky (4.5%), feeling it was too uncomfortable (27.3%), and not being allowed to carry it (4.5%).

**Table 5:** Contextual characteristics of EMA-reported sports or exercise.

Characteristic		n (%)^b^
Social context		
	Alone	9 (22.0)
	Not alone	32 (78.0)
Physical context		
	Outdoors	30 (69.8)
	Indoors	13 (30.2)
Elevation change		
	Mainly staying on flat ground	38 (76.0)
	Mainly going uphill	4 (8.0)
	Mainly going downhill	4 (8.0)
	Going both uphill and downhill	4 (8.0)
Purpose		
	For work, homework, or housework	1 (2.2)
	Fun or recreation	28 (62.2)
	Personal care	5 (11.1)
	To get somewhere	2 (4.4)
	Other	9 (20.0)
	Baby sitting or childcare	0
Load bearing		
	More than 20 lbs	3 (7.3)
	10-20 lbs	1 (2.4)
	5-10 lbs	4 (9.8)
	Less than 5 lbs	3 (7.3)
	None	20 (48.8)
Exercise form^a^		
	None	3 (8.6)
	Flexibility	19 (54.3)
	Endurance	25 (71.4)
	Balance	13 (37.1)
	Strengthening	0
Type of exercise or sport		
	Basketball, football, or soccer	16 (15.1)
	Bicycling	3 (2.8)
	Exercise, dance, or karate class	15 (14.2)
	Other sports (baseball, skateboarding, and so forth)	27 (25.5)
	Other running or jogging	20 (18.9)
	Swimming	1 (0.9)
	Walking	16 (15.1)
	Weightlifting or strength training	8 (7.6)

^a^Participants could select more than one form of exercise.

^b^n=35 total responses.

Table 5 summarizes contextual and situational characteristics of sports and exercise reported through R-EMA + CS-EMA. The most common type of exercise or sport reported was *Other sports,* followed by *Other running/jogging, Basketball/Football/Soccer*, and *Walking*. Children most frequently reported that the purpose of performing the sport or exercise was for *Fun/Recreation*, and the most common form was endurance activities. Most children did not carry any extra weight during the sport or exercise, but a small percentage reported carrying over 20 lbs. Almost three-quarters of the activity occurred on flat ground (ie, not going uphill or downhill). Over two-thirds of the sports and exercise activities occurred outdoors, and a quarter occurred alone.

## Discussion

Children recruited into surveillance, epidemiological, and intervention studies will increasingly have mobile phones, which are miniature computers with built-in motion sensors and electronic survey administration capabilities. Mobile phones are becoming more ubiquitous, affordable, and easy to use. The mobile phones are rarely far from the adolescents, and their affinity for the mobile phones creates new opportunities for real-time monitoring. This study tested the feasibility, performance, and utility of the Mobile Teen EMA app for capturing information about physical activity behaviors and contexts in adolescents.

Overall, participants answered over 80% of EMA surveys that were prompted, of which over 80% were answered after the first prompt. Relative to prior EMA work, this compliance could be considered moderate to high [27,28], especially because participants had the option to mute the mobile phone’s audio. However, about a quarter of scheduled EMA surveys were never prompted because the mobile phone was either powered off or experienced an unknown technical problem. It is not clear whether participants intentionally turned off their mobile phones because they did not want to receive EMA prompts during those times, or whether the mobile phone battery died. Either way, EMA studies should make efforts to encourage participants to keep mobile phones powered on and fully charged during the hours of the days when EMA prompting is occurring, and EMA apps that use the mobile phone’s sensing capabilities will need to be designed so that the sensing does not lead to additional battery drain and subsequent data loss. On average, EMA question sequences required less than 1 minute to complete, and participants completed surveys 20 seconds faster at the end of the 14-day monitoring period than at the beginning of the study—indicating improved ease and comfort with the survey items and procedures with practice. Furthermore, participants fully completed (ie, answered all questions) over 98% of the EMA surveys they started.

Adolescents may have been more likely to respond to CS-EMA than R-EMA prompts because CS-EMA prompts were triggered by a change in the state of the mobile phone device (eg, increase or decrease in movement), indicating that the participant was interacting with the mobile phone. In contrast, R-EMA may have been more likely to occur at times when the adolescent was away from the mobile phone (eg, sleep, bathing). The decreasing EMA prompt compliance rate across the 14-day monitoring period, however, suggests that some participant fatigue or growing disinterest may have occurred. Moreover, the lower EMA compliance rate in the mornings as compared with later times in the day may reflect participants’ inability (due to sleep) or reluctance to answer EMA prompts in the early mornings on weekends. Girls may have been less likely to carry the mobile phones on their bodies than boys because the mobile phones did not fit as well in their pant pockets. Because girls are less likely to carry the mobile phone so that it has physical contact with the body, the built-in mobile phone accelerometers may be less likely to capture their fine-grained body movement patterns. However, the mobile phone may still have had the ability to detect major transitions in body movement patterns if the mobile phone was usually *“Within reach,”* as was reported (eg, sitting on a table nearby), which would suggest that girls tended to carry it with them when they make transition in location (even when indoors).

A second objective of this study was to evaluate the performance of the sensor-informed CS-EMA prompt triggers by examining differences in reported and objectively measured activity levels across the different EMA prompt types. The fact that levels of physical activity independently detected by the waist-worn accelerometer activity monitor were significantly higher during the time periods immediately leading up to CS-EMA Activity type prompts as compared with all other types of EMA prompts provides further support that full body movement (not just mobile phone movement) was indeed elevated during those periods. However, it is important to note that CS-EMA prompts did not capture all physical activity episodes that occurred. The CS-EMA Activity threshold was likely lower than the MVPA threshold applied to waist accelerometer data, as evidenced by most CS-EMA Activity prompts no more than light physical activity. Furthermore, participants were likely not in possession of their mobile phones during MVPA activities lasting longer than 15 minutes, and the CS-EMA No-Activity detection period was too long to capture this period of time because there were no bouts of MVPA longer than 60 minutes. Sports and exercise were also reported during CS-EMA No-Activity and CS-EMA No-Data type prompts. These findings are not surprising in light of the fact that during reported sports and exercise bouts, adolescents indicated that mobile phones were *“Within reach”* (but not being worn on the participant or not with the participants) over 30% of the time. Although CS-EMA Activity prompts may miss some sports and exercise owing to noncarrying of the mobile phone by adolescents, these bouts may be captured instead by the CS-EMA No-Activity and CS-EMA No-Data type prompts when the participants return to their mobile phones or turn them on again after physical activity. It is interesting to note that *“Going somewhere* ” was 5 times as likely to occur before CS-EMA Activity prompts compared to R-EMA prompts, suggesting that CS-EMA Activity type prompting may be a good method to capture physically active travel among adolescents (eg, walking or bicycling for transportation).

The third and fourth objectives of this study address the utility of CS-EMA informed by onboard mobile phone motion sensors in providing information about physical activity and sedentary behavior that would not otherwise be captured through a waist-worn accelerometer alone. One of the benefits of EMA is that it has the potential to yield activity data during periods when the waist-worn accelerometer is not being worn. Most adolescents are highly motivated to carry and keep charged and operate their own personal mobile phones. The internal motion sensor data from the mobile phones should therefore capture major transitions throughout the day, regardless of whether the waist-worn accelerometer is being worn. Even when an adolescent fails to have either device (waist-worn accelerometer or mobile phone) on his or her body, such as during high-contact sports and swimming, the CS-EMA feature should automatically trigger a CS-EMA No-Activity or CS-EMA No-Data survey prompt immediately after the adolescent begins carrying or using the mobile phone again. Thus, the CS-EMA may capture information about exercise or sports even if the mobile phone is not carried during these activities. Data about the type and duration of activities performed collected through CS-EMA can be used to estimate energy expenditure during accelerometer nonwear periods. Activity categories selected through CS-EMA reporting what the participant did during accelerometer nonwear periods can be converted to METs using the Compendium of Physical Activities [29] and multiplied by the duration of known device nonwear (in minutes) to generate an estimate of energy expenditure (in MET∙minutes) for that period of time. These energy expenditure estimates can then be imputed to fill nonwear holes in objective activity data to obtain a more accurate representation of levels of physical activity and sedentary behavior across that day. In this study, EMA-reported activity type data were provided during about 32 minutes per day of waist-worn accelerometer nonwear.

Additional information about physical activity episodes provided by EMA that would not otherwise be captured through a waist-worn accelerometer includes situational and contextual characteristics. Data from the sensor-informed EMA can be used to improve energy expenditure estimates for activities not well captured by waist-worn motion sensors, such as those that involve the upper body (eg, weightlifting or strength training was reported in almost 8% of sports or exercise bouts), cycling (reported in almost 3% of sports or exercise bouts), weight bearing (some weight reported being carried on over 50% of sports or exercise bouts), and incline or decline (reported in almost 25% of sports or exercise bouts). These data can be used to upwardly or downwardly adjust energy expenditure estimates obtained from objective activity monitors [11,30]. Furthermore, EMA data may also be used to differentiate between conceptually distinct activity types (eg, exercise, dance, or karate class [14% of sports or exercise bouts] vs running or jogging [19% of sports or exercise bouts]), which may appear identical when examining objective activity intensity data alone. In addition, EMA gathers data about the purpose of the activity (eg, fun or recreation [62% of sports or exercise bouts], to get somewhere [4% of sports or exercise bouts], for work or housework [2% of sports or exercise bouts]) that may be useful in assessing the amount of transit- and work-related physical activity performed. Finally, the EMA questions gather information about where, with whom, and why physical activity occurs, as well as how participants feel during those activities. These data help researchers to understand whether physical activity intensity or duration differs across contexts [31] and to investigate time-varying antecedents and consequences of behavior [27].

One possible concern with the method as proposed is that the Mobile Teen app depends on adolescents in future activity measurement studies using personal mobile phones. The mobile phones they have may not be appropriate mobile phones for running the Mobile Teen app. In those cases, some of the adolescents could be switched to appropriate mobile phones by temporarily swapping SIM cards, as was done in the Mobile Teen testing (for approximately 20% of participants). The technology in its current form will only work on Android mobile phones because iOS will not support the required background processing, but over 80% of new mobile phone shipments worldwide use Android [32], and changes to Apple’s iPhone line adding a motion coprocessor chip may allow continuous movement measurement [33] and thereby create opportunities to develop versions of Mobile Teen for new iPhones as well. CS-EMA activity and duration thresholds could also be modified in future iterations to better capture episodes of physical activity for a given population, especially when the participant is not in possession of a mobile phone. It should be noted that the assessment time frame used in this study (3-9 pm on weekdays and 7 am-9 pm on weekend days) did not permit an overall level of physical activity to be measured (because physical activity taking place on the way to school or during physical education classes was not included). Another concern often raised with EMA is reactivity, the potential for behavior to be impacted by the very act of assessing it, but the magnitude of reaction to EMA has been observed to be small for EMA studies [34-36]. Another limitation is the ~20% of prompted EMA surveys that are unanswered, which are more common in the mornings. Reasons for lower compliance rates in non-Hispanic (vs Hispanic) children are largely unknown and should be explored in future research. However, it should be noted that Hispanic children comprised the majority (66%) of the current sample. There may be other unmeasured variables that correlate with noncompliance to EMA prompting such as negative mood and stress, which should be explored in future studies. Furthermore, this study tested the Mobile Teen app on a relatively small sample of primarily Hispanic adolescents from an urban Los Angeles high school. Further testing is needed in larger samples of adolescents from other regions of the United States and internationally.

Ultimately, the sensor-informed CS-EMA methods used by the Mobile Teen app can be used to augment and supplement physical activity data collected through external objective activity monitors (such as waist or wrist-worn accelerometers). Studies may deploy CS-EMA procedures in a stand-alone manner or in conjunction with objective activity monitoring, depending on which characteristics of physical activity are desired for assessment. For example, if a study seeks to understand the types (and contexts) of physical activity that result in bouts of high-intensity activity that last 15+ minutes, sensor-informed CS-EMA procedures could be used alone without an external objective activity monitor. In addition, sensor-informed CS-EMA may be used together with an external objective activity monitor to capture and estimate energy expenditure during times when the external objective activity monitor is not worn. Furthermore, the sensor-informed CS-EMA procedures described in this study can provide the basic architecture for Just-In-Time Adaptive Interventions (JITAIs) [12,37,38], targeting physical activity change. JITAIs use real-time decision rules to link a participant’s current situation, sometimes inferred from sensor data, with appropriate, tailored intervention strategies intended to have optimal impact. Sensor-informed CS-EMA procedures can be built into the learning phases of JITAIs to collect information about affective, motivational, and contextual factors that are ideographically related to naturally occurring physical activity variations and inform the development of intervention content and messages. Sensor-informed CS-EMA can also guide the timing during the subsequent intervention delivery phase of JITAIs by prompting individuals to break up elongated bouts of sedentary behavior or to lengthen short bouts of higher intensity physical activity detected by the mobile phone. The opportunity to move beyond randomly delivered behavior change messages to an informed and timely delivery strategy has enormous potential benefits in the intervention domain.

## Abbreviations

BMI: body mass index
BS: between subjects
EMA: ecological momentary assessment
CI: confidence interval
CS-EMA: context-sensitive ecological momentary assessment
GSM: global system for mobile communication
JITAI: Just-In-Time Adaptive Intervention
MET: metabolic equivalent
MVPA: moderate-to-vigorous physical activity
OR: odds ratio
R-EMA: random ecological momentary assessment
SD: standard deviation
SE: standard error of the mean
SIM: subscriber identity module
SMS: short message service
WS: within subjects
